# An intervention to reassure patients about test results in rapid access chest pain clinic: a pilot randomised controlled trial

**DOI:** 10.1186/1471-2261-14-138

**Published:** 2014-10-04

**Authors:** Kathryn Hicks, Kim Cocks, Belen Corbacho Martin, Peter Elton, Anita MacNab, Wendy Colecliffe, Gill Furze

**Affiliations:** Department of Health Sciences, York Trials Unit, University of York, York, YO10 5DD UK; Greater Manchester, Lancashire & South Cumbria Strategic Clinical Network, 4th Floor, 3 Piccadilly Place, Manchester, M1 3BN UK; University Hospital of South Manchester, North West Heart Centre, Southmoor Road, Manchester, M23 9LT UK; Faculty of Health & Life Sciences, Coventry University, Richard Crossman Building, Priory Street, Coventry, CV1 5FB UK

**Keywords:** Reassurance, Rapid access chest pain clinic, RACPC, Pilot study, Randomised controlled trial, Angina, Coronary heart disease, Ischaemic heart disease, Non-cardiac chest pain, Brief intervention

## Abstract

**Background:**

Most people referred to rapid access chest pain clinics have non-cardiac chest pain, and in those diagnosed with stable coronary heart disease, guidance recommends that first-line treatment is usually medication rather than revascularisation. Consequently, many patients are not reassured they have the correct diagnosis or treatment. A previous trial reported that, in people with non-cardiac chest pain, a brief discussion with a health psychologist before the tests about the meaning of potential results led to people being significantly more reassured. The aim of this pilot was to test study procedures and inform sample size for a future multi-centre trial and to gain initial estimates of effectiveness of the discussion intervention.

**Methods:**

This was a two-arm pilot randomised controlled trial in outpatient rapid access chest pain clinic in 120 people undergoing investigation for new onset, non-urgent chest pain. Eligible participants were randomised to receive either: a discussion about the meaning and implication of test results, delivered by a nurse before tests in clinic, plus a pre-test pamphlet covering the same information (Discussion arm) or the pre-test pamphlet alone (Pamphlet arm). Main outcome measures were recruitment rate and feasibility for a future multi-centre trial, with an estimate of reassurance in the groups at month 1 and 6 using a 5-item patient-reported scale.

**Results:**

Two hundred and seventy people attended rapid access chest pain clinic during recruitment and 120/270 participants (44%) were randomised, 60 to each arm. There was no evidence of a difference between the Discussion and Pamphlet arms in the mean reassurance score at month 1 (34.2 vs 33.7) or at month 6 (35.3 vs 35.9). Patient-reported chest pain and use of heart medications were also similar between the two arms.

**Conclusions:**

A larger trial of the discussion intervention in the UK would not be warranted. Patients reported high levels of reassurance which were similar in patients receiving the discussion with a nurse and in those receiving a pamphlet alone.

**Trial registration:**

Current Controlled Trials ISRCTN60618114 (assigned 27.05.2011).

**Electronic supplementary material:**

The online version of this article (doi:10.1186/1471-2261-14-138) contains supplementary material, which is available to authorized users.

## Background

Chest pain is a common reason why people access health services, with over 400,000 people per year being referred to rapid access chest pain clinics (RACPCs) in England [1]. RACPCs were set up in the UK to assess patients with new onset chest pain within two weeks of reporting symptoms to their general practitioner (GP) [2]. In clinic patients undergo basic clinical assessment and investigation in order to confirm or rule out coronary heart disease (CHD) as a cause of their chest pain. Although the main focus in RACPC is detection of new angina, the majority (up to 80%) of patients will be categorised as having non-cardiac chest pain (NCCP) [3–5]. However, as studies in people undergoing outpatient tests for heart disease have shown, many with a negative (normal) result are not reassured that their chest pain is non-cardiac in origin [6, 7], and continue to report chest pain in the following months and use NHS services [3, 8]. There are several causes of NCCP, including physical problems (e.g. gastroesophageal disorders, musculoskeletal causes) or psychological disorders (such as anxiety, panic attacks, and depression), and often there is an interaction between psychological and physical causes [9].

Psychological factors have been targeted for treatment of NCCP. A recent review of randomised controlled trials (RCTs) of psychological interventions for symptomatic management of non-specific chest pain in patients with normal coronary anatomy showed modest to moderate success in terms of reduced chest pain frequency, particularly for those using cognitive behavioural therapy [10]. This success was relatively short term, being largely restricted to the 3 months after the intervention. Brief interventions, delivered immediately after negative (normal) findings, have been tested but these have mostly been unsuccessful. For example, an RCT of a brief psychological intervention for people following coronary angiography who were told they had normal coronary arteries showed no benefit [11]. The authors concluded that the patients were “clearly ill prepared for the possibility of negative findings”.

Petrie et al. (2007) undertook a small RCT in New Zealand (NZ) to assess whether it was possible to improve people’s preparedness for negative results, and so increase reassurance [12]. The study compared usual care with two interventions delivered before tests in chest pain clinic, as Petrie et al. hypothesised that providing a pre-test explanation about the meaning of normal test results would weaken preconceptions about possible illness, provide context for the results and so increase the person’s potential for reassurance. The interventions were: i) a pamphlet giving information about the meaning of normal results and other possible reasons for chest pain, and ii) the pamphlet *plus* a brief pre-test discussion with a health psychologist re-iterating the same information. People receiving the pre-test discussion were more reassured at one month after the test than patients randomised to the pamphlet alone or to the usual care arm (results explained after the test). Only the difference between the discussion and usual care arms reached statistical significance when considering mean reassurance score (from a 5-item patient-reported scale). The proportion of patients reporting chest pain at one month decreased significantly from baseline in the discussion group and pamphlet-only group, but not in the usual care group. It is possible that a key element in the relative success of the NZ pre-test interventions was the fact that they were delivered before the test. Donkin et al. found that, in people with NCCP, patients’ beliefs before an exercise stress test predicted the amount of reassurance they felt once they had received their negative test result [13].

It is not only those who get a negative result in RACPC that may require reassurance; patients who receive a positive result (CHD) may also need reassuring. According to guidance from the National Institute for Health and Care Effectiveness (NICE), initial treatment for people diagnosed with stable CHD should be optimal pharmacotherapy to control symptoms together with effective secondary prevention. Interventions such as percutaneous coronary intervention or coronary artery bypass graft surgery should be restricted to those in whom optimal medical treatment fails to reduce symptoms, or if further testing by non-invasive imaging or by angiography shows left main stem or severe three-vessel disease [14]. This pathway needs to be explained well to patients in order to reassure them that they are receiving the optimal treatment for their condition. For example, it is possible that patients prescribed medical treatment rather than invasive revascularisation may feel that they are receiving a second-best treatment. The converse is also true; people referred for invasive intervention will need to be reassured that this level of treatment is appropriate for them. A relationship between satisfaction with treatment and reports of anxiety, depression and quality of life has been demonstrated [15], hence dissatisfaction with treatment may lead to an increase in health service use.

The NZ study was limited by small sample size and short duration of follow-up (one month), and it only included people with NCCP. The clinical pathway for patients with chest pain differs between NZ and the UK and the discussion intervention was delivered by a health psychologist, a profession which is not routinely available in NHS outpatient clinics.

The aim of this study was to adapt the discussion intervention for delivery in UK RACPCs and conduct a 2-arm pilot RCT comparing discussion (plus pamphlet) versus pamphlet alone. The pre-test pamphlet had already been recommended for use at the study site, as part of the chest pain pathway. The aim of the pilot trial was to test study procedures and gain data to inform the choice of primary outcome measure and the sample size calculation for a future, multi-centre RCT of effectiveness and cost-effectiveness. A preliminary investigation into whether the face-to-face discussion with a nurse may improve patient reassurance was also made. If a simple discussion intervention was found to be effective and cost-effective when delivered by a nurse to people with both NCCP and with a diagnosis of CHD in UK RACPCs, then it could be relatively easily incorporated into the clinical pathway.

## Methods

### Development of the interventions

The NZ discussion intervention and pamphlet were adapted so that they covered both positive and negative test results, and the different treatment options. This was undertaken by an expert reference group with input from service users and RACPC staff. The pamphlet (A5, 4-page booklet, 664 words; see Additional file 1) outlined: the three possible results from tests in RACPC that day (negative/normal (NCCP), positive/abnormal (CHD), or inconclusive i.e. need to return for more tests); the meaning of negative results with a high risk of developing heart disease versus low risk; possible reasons for chest pain in those with a negative result (e.g. muscular, gastroesophageal reflux disease); what to do if results are negative but chest pain continues; and treatment options for those with a positive result (medication with review at 3 months or angiogram, possibly indicating angioplasty or surgery). The brief discussion intervention (5–15 min), to be delivered by a research nurse rather than a health psychologist, re-iterated the same information, and checked that the patient understood the information. A topic guide (463 words) was developed for the research nurse delivering the intervention. The guide was not a script to be read verbatim, but outlined the topics to be covered in the discussion. The research nurse was trained by the Chief Investigator to deliver the discussion intervention and to check that the patient understood the information.

### Study design and setting

This was a single-centre, two-arm, pilot RCT comparing a pre-test discussion intervention plus a pre-test pamphlet (covering the same information) versus the pamphlet alone in patients attending RACPC with new onset chest pain. The study was conducted at the University Hospital of South Manchester (UHSM; Manchester, UK) RACPC. It was approved by North West 9 Research Ethics Committee – Greater Manchester West (reference no. 10/H1014/82) and the R&D Directorate of UHSM NHS Foundation Trust.

### Study population and patient consent

Patients were sent information about the trial with their RACPC appointment letter and asked on arrival at clinic if they wished to take part. Patients were eligible if they were: attending RACPC for assessment of new-onset, non-urgent chest pain; able to read written English; able to comprehend spoken English; aged 18 years and over and able and willing to give informed consent. Patients were excluded if they: had a previously diagnosed cardiac pathology; had no symptoms of chest pain; were undertaking the exercise test as part of a pre-surgical medical examination; were pregnant; were involved in another research study; had a severe documented psychiatric disorder or had a life-threatening co-morbidity. Eligible patients gave written informed consent.

### Randomisation

Patients were randomised by a research nurse telephoning a remote randomisation service (York Trials Unit (YTU), University of York, UK). Random permuted blocks (block sizes of four and six) were used to allocate patients in a 1:1 ratio.

### Blinding

Patients were told that the study was to compare two different ways of giving information about possible test results and treatments but not what the two formats were (pamphlet and discussion), or that one (the pamphlet) was considered to be the control arm. Staff treating patients in the RACPC appointment were not informed of treatment group allocation.

### Intervention arms

After randomisation, all patients were given the pre-test pamphlet by the research nurse and were allowed sufficient time to read it. Patients allocated to the Discussion arm were then engaged in a brief (5–15 min) discussion with the research nurse. After receiving the pamphlet and discussion (Discussion arm), or the pamphlet alone (Pamphlet arm), patients returned to clinic and underwent assessment and tests as usual. All patients received the usual advice and information from staff during the initial RACPC appointment and any visits for further tests.

### Assessment in RACPC

Assessment in clinic was led by a senior cardiac specialist nurse according to UHSM’s protocol. It included an assessment of risk factors, typicality of symptoms (symptom score 0 to 3), Diamond and Forrester percentage score [16] and the probability that the patient had a cardiac cause for the pain (high, medium or low). Patients had the following tests as indicated: resting electrocardiogram, blood pressure measurement, auscultation of heart sounds, blood test for lipid profile and/or other analyses, exercise tolerance test (ETT), dobutamine stress echocardiogram (DSE), exercise echocardiogram, myocardial perfusion scan (MPS) and magnetic resonance imaging (MRI). The assessment in RACPC lasted up to four hours depending on the number of tests conducted.

At the end of the initial appointment patients were either given a diagnosis (CHD or NCCP) or, if a particular test (usually DSE) could not be performed on the day, they were asked to return for further tests. In each case, test results/diagnosis were explained to the patient by a clinic nurse, face-to-face, before leaving clinic. Patients with NCCP were reassured that there was no evidence of coronary disease and were advised to see their GP within the next week for reassessment for other causes of the chest pain. Other possible causes of the chest pain were suggested if the patient asked, but the nurse emphasised that they should go to their GP. If patients were at high risk of developing heart disease, lifestyle-change advice and leaflets were provided to the patient, and their GP was informed that aggressive risk factor management was needed. A CHD diagnosis resulted in the initiation of appropriate treatment: medication with review after 3 months or referral for an angiogram (possibly followed by angioplasty or surgery).

### Outcome measurements

As this was a pilot study, primary outcomes included recruitment rate and process, proportion of patients attending RACPC randomised, and reasons for non-participation. The study was powered to give initial estimates of effectiveness using the patient-reported 5-item reassurance questionnaire used in the NZ study [12] (Table 1). Outcomes were proportion of patients reassured (as defined by Petrie et al.) and reassurance score at month 1 and month 6. The validity/reliability of the 5-item reassurance questionnaire in this population was assessed.

**Table 1:** **5-item reassurance questionnaire**

1. How worried are you about your health?										
0	1	2	3	4	5	6	7	8	9	10
Not at all										Extremely worried
2. How much do you believe that there is something seriously wrong with your heart?										
0	1	2	3	4	5	6	7	8	9	10
Not at all										Strongly believe
3. How reassured were you by the test?										
0	1	2	3	4	5	6	7	8	9	10
Not at all										Extremely reassured
4. How much do you believe that you will need further tests to find out the cause of your illness?										
0	1	2	3	4	5	6	7	8	9	10
Not at all										Strongly believe
5. How accurate do you think the test was for identifying heart problems?										
0	1	2	3	4	5	6	7	8	9	10
Not at all										Extremely accurate

The three negatively worded items (1, 2 and 4) were reversed and the five scores summed, a higher score indicating higher levels of reassurance.

Secondary outcomes included the feasibility/acceptability of the discussion intervention, follow-up rates, questionnaire completion rates, and patient-reported chest pain, heart-related drug use, Hospital Anxiety and Depression Scale (HADS) [17], Brief Illness Perception Questionnaire (BIPQ) [18], Seattle Angina Questionnaire - UK version (SAQ-UK) [19] with reference to chest pain/tightness retained but reference to angina removed, Guys and St Thomas’ chest pain questionnaire [20], EQ-5D [21] and NHS resource use for chest pain (GP visits; inpatient, out-patient and emergency hospital visits). Patients completed questionnaires at baseline (collected by research nurse prior to randomisation), at the end of the RACPC appointment (“post-clinic”; 5 reassurance questions only; completed prior to leaving clinic), at month 1 and at month 6, and a 7-day chest pain diary at month 1 and month 6 (returned to YTU by post).

### Sample size

As a pilot trial, the main aim was to inform the feasibility and sample size of a future multi-centre trial, however a sample size calculation was carried out in order to obtain reasonable estimates of effectiveness within the study. Petrie et al. [12] observed a difference between the discussion and pamphlet-alone arms with respect to the proportion of patients reassured at one month (69% versus 40% respectively). Assuming similar proportions for this study, 120 patients would be required to detect a difference with 80% power for a 2-sided, 5% significance level, allowing for a 20% drop out rate.

### Statistical methods

All analyses were conducted on an intention to treat basis, including all patients in the arms to which they were randomised, assuming data were available. No imputation was carried out in this pilot analysis. Analyses were conducted in SAS version 9.3 (SAS Institute, NC, USA).

The reliability and validity of the 5-item reassurance questionnaire was investigated using standard psychometric tests. Internal consistency between items was tested using Cronbach’s alpha, where >0.7 was considered acceptable [22]. The distribution of individual items was summarised in bar graphs to assess for skewness and floor or ceiling effects. An assessment of test-retest reliability was planned using the subset of patients with negative results (NCCP diagnosis) at the end of their RACPC appointment (post-clinic). We hypothesized that the post-clinic and month 1 assessments would be stable for these patients. Known group comparisons (using t-tests) were planned between CHD and NCCP patients and between patients with low and high anxiety at baseline to see if the questionnaire could distinguish between these groups of patients where reassurance would be expected to be different.

The proportion of patients reassured (defined as above the median reassurance score, as per Petrie et al. [12]) was summarised by intervention arm and timepoint. Since the categorisation using the median is fairly arbitrary we considered the continuous reassurance score as primary. We also investigated whether the categorisation above and below the median resulted in classifications as reassured and not reassured broadly in line with how patients answered item 3 (How reassured were you by the test?). Reassurance scores were compared between arms using a repeated measures mixed model, accounting for the baseline reassurance questions. No formal statistics were planned or performed for any of the secondary outcomes in this pilot study. Patient-reported outcomes were summarised by intervention arm and timepoint. The internal consistency of the overall BIPQ score was tested using Cronbach’s alpha, where >0.7 was considered acceptable.

### Health economic data

Patient-reported data on resource use (from the perspective of the NHS and Personal Social Services) and health related quality of life (EQ5D) were summarised by intervention arm and timepoint in order to preview future cost-effectiveness analysis issues, such as the accuracy and completeness of the data collection methods and the need to account for censored/missing data in a future trial.

## Results

### Primary outcomes

#### Recruitment and patient sample

**Figure 1:**
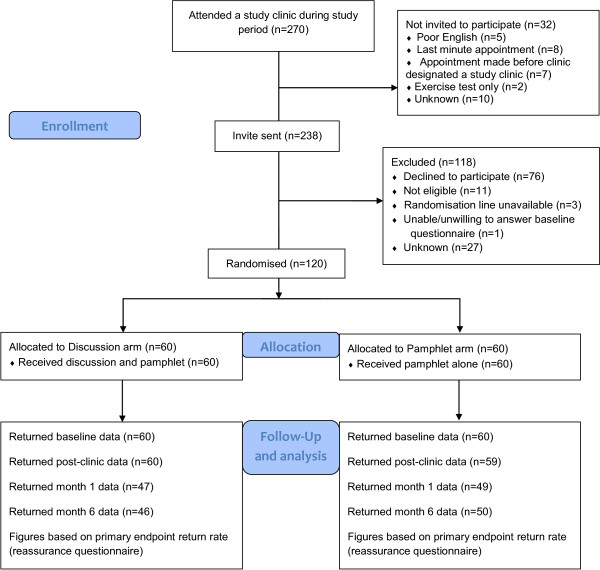
**Flow of participants through the study.**

120 patients (60/arm) were recruited in 8 months (Oct 2011 to May 2012). Figure 1 shows the flow of participants through the study and Table 2 summarises their baseline characteristics. Around 40% of patients attending RACPC were randomised in the study. All participants received their allocated intervention: the pre-test pamphlet plus a discussion with a nurse (Discussion arm) or the pre-test pamphlet alone (Pamphlet arm). Patients had a mean age of 54 (SD 12) and just under half were male. Sixty-two (52%) patients were taking heart-related medications at baseline, the majority of which were from one or more of the following classes: beta blockers, statins, anti-platelets, glyceryl trinitrate, other anti-anginals.

**Table 2:** **Baseline characteristics of the study population**

Patients N (%)	Allocation	
	Discussion (n = 60)	Pamphlet (n = 60)
Male:Female	29 (48%): 31 (52%)	30 (50%): 30 (50%)
Mean age (years) (SD)	55 (12.3)	53 (12.0)
Ethnicity:		
White	52 (87%)	51 (85%)
Mixed	2 (3%)	1 (2%)
Asian or Asian British	5 (8%)	7 (12%)
Black or Black British	1 (2%)	0 (0%)
Other	0 (0%)	1 (2%)
Education beyond minimum school leaving age	33 (55%)	41 (68%)
Degree or equivalent qualification	23 (38%)	27 (45%)
Employment:		
Employed/Self-employed	32 (53%)	35 (58%)
Retired	12 (20%)	13 (22%)
Other	13 (22%)	12 (20%)
Marital Status:		
Single	8 (13%)	14 (23%)
Married/permanent partnership	41 (68%)	33 (55%)
Divorced	4 (7%)	9 (15%)
Widowed	7 (12%)	4 (7%)
Number of weeks with chest pain (median, range)	6 (1–500)	6 (1–200)
Number of times in last 7 days had chest pain (median, range)	3 (0–14)	2 (0–21)
Taking heart-related* medications at baseline	30 (50%)	32 (53%)
High risk for CHD	32 (53%)	34 (57%)

*Heart-related medications included beta blockers, statins, anti-platelets, glyceryl trinitrate and other anti-anginals.

*SD* standard deviation, *CHD* coronary heart disease.

Forty-three (35.8%) patients received a diagnosis at their initial clinic visit, whilst 65 (54.2%) returned for further tests and got their diagnosis at a second visit. The remaining 12 (10.0%) patients were invited to return for further tests but declined. Cardiac tests performed are presented in Table 3. Final diagnoses are presented in Table 4, with 76% of patients being diagnosed with NCCP. Time to diagnosis from initial RACPC visit was a median of 7 days (range 0 to 181 days), with 75% of patients receiving their diagnosis within two weeks of their initial RACPC visit.

**Table 3:** **Cardiac tests performed by intervention arm**

	Discussion (n = 60)	Pamphlet (n = 60)	Total (n = 120)
Exercise tolerance test (ETT)	29	38	67
Dobutamine stress echocardiogram (DSE)	28	24	52
Exercise echocardiogram	5	8	13
Echocardiogram	1	0	1
Myocardial perfusion scan (MPS)	0	0	0
Magnetic resonance imaging (MRI)	0	0	0

**Table 4:** **Test results by intervention arm**

Diagnosis	Allocation				All	
	Discussion		Pamphlet			
	N	%	N	%	N	%
CHD	10	16.7	7	11.7	17	14.2
*- Referred for angiogram*	*3*		*4*			
*- Medication with review at 3 months*	*7*		*3*			
NCCP	46	76.7	45	75.0	91	75.8
Inconclusive	4	6.7	8	13.3	12	10.0

*CHD* coronary heart disease, *NCCP* non-cardiac chest pain.

#### Reassurance questionnaire validity

Return rates for the reassurance questionnaire were good, with 80% of participants returning a questionnaire at month 1 and at month 6. However, the timing of questionnaire return varied considerably, with month 1 questionnaires being completed at a median of 6 weeks (range 4 to 17) and month 6 questionnaires at a median of 28 weeks (range 24 to 41).

The validity of the reassurance questionnaire was investigated in this sample as previously it was used for NCCP patients only [12]. Although there was some skewness, the plots of responses to individual items did not show any problems with floor or ceiling effects (see Additional file 2). Internal consistency was borderline at the post-clinic timepoint (Cronbach’s alpha = 0.68) and acceptable at months 1 and 6 (0.82 and 0.78 respectively). Test-retest reliability could not be established as, in practice, the time between the post-clinic and planned month 1 questionnaire was too long to be considered a stable period (average 6 weeks). The subset of patients with questionnaires within a two week window around month 1 showed significant correlation between the two timepoints although patient numbers were too small (n = 11) to conclude test-retest reliability. Known group comparisons showed the questionnaire could distinguish between clinically distinct groups of patients, i.e. those with CHD versus NCCP and those with high versus low anxiety.

#### Proportion reassured and reassurance score

**Figure 2:**
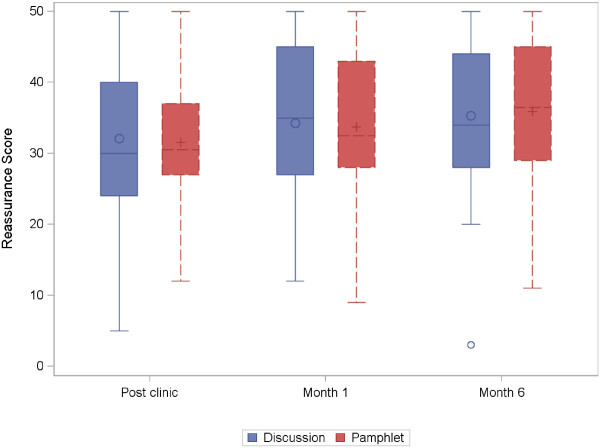
**Reassurance score over time.** Score ranges from 0 to 50 with higher scores representing more reassurance. The median is represented by a line and the mean by 'O' or ' + '. The shaded box represents the interquartile range. Outliers are identified using 'o'.

Differences between the arms in the proportion of patients classed as reassured were not consistent over time (53% vs 48% at month 1 and 49% vs 58% at month 6 in the Discussion and Pamphlet arms respectively; Table 5). Mean reassurance scores across the Discussion and Pamphlet arms were similar at all timepoints; p = 0.08 using a repeated measures model (Figure 2). Question 3 (How reassured were you by the test?) indicated reasonably high reassurance immediately post-clinic and through to month 6 in both arms (mean score 7 or 8 for each arm at post-clinic, month 1 and month 6).

**Table 5:** **- Proportion of patients reassured at post-clinic, month 1 and month 6**

% (95% confidence interval)	Post-clinic (n = 117)	Month 1 (n = 95)	Month 6 (n = 95)
Discussion	50.9 (38.1 to 63.6)	53.2 (38.9 to 67.5)	48.9 (34.3 to 63.5)
Pamphlet	58.6 (46.9 to 71.3)	47.9 (33.8 to 62.1)	58.0 (44.3 to 71.7)

### Secondary outcomes

#### Follow-up rates and questionnaire completion rates

There were no patient withdrawals or change of circumstances notified to the study site or YTU during the study. Completion rates were highest for the primary outcome (reassurance questionnaire; 79-99%). Other questionnaires ranged from 28-100% completed (Table 6). Results presented as proportions of patients in the following sections use these completion rates as the denominator unless otherwise stated.

**Table 6:** **Questionnaire completion rates**

Number (%) completed (of 120 randomised)	Baseline	Post-clinic	Month 1	Month 6
Reassurance questionnaire	119 (99%)	117 (98%)	95 (79%)	95 (79%)
Chest pain questions	113 (94%)	NA	92 (77%)	92 (77%)
Chest pain diaries	NA	NA	88 (73%)	74 (62%)
Heart-related medications	120 (100%)^*^	NA	97 (81%)	98 (82%)
BIPQ (overall score)	98 (82%)	NA	72 (60%)	61 (51%)
SAQ-UK	119 (99%)	NA	92 (77%)	80 (67%)
HADS	117 (98%)	NA	92 (77%)	83 (69%)
Guys and St Thomas	91 (76%)	NA	56 (47%)	33 (28%)
EQ5D	115 (96%)	NA	90 (75%)	82 (68%)
NHS resource use	NA	NA	91 (76%)	83 (69%)

NA = Not applicable at this timepoint.

*Collected by clinic staff at initial RACPC visit; all other questionnaires were patient-reported.

#### Patient-reported chest pain

At baseline 92/113 (81%) patients reported some chest pain in the previous seven days. At month 1 a smaller proportion of the Discussion arm reported chest pain in the period since they last completed a questionnaire, 24/45 (53%) compared to 29/47 (62%) of the Pamphlet arm. At month 6 these proportions were more similar (59% in the Discussion arm vs 52% in the Pamphlet arm).

Data from a 7-day chest pain diary completed at month 1 and month 6 showed a similar reduction in the proportion of patients reporting chest pain over time in both arms (see table in Additional file 3).

#### Heart-related medications

52% of patients were taking heart-related medication at baseline and the proportion reduced over time in both arms. A similar proportion in both arms reported taking heart-related drugs at month 1 (43% in the Discussion arm and 38% in the Pamphlet arm). At month 6 the proportions were 40% of the Discussion arm and 29% of the Pamphlet arm.

#### Other secondary outcomes

The discussion intervention, assessed through qualitative interviews, was found to be acceptable to patients and staff. This will be reported more fully elsewhere. Change from baseline in treatment satisfaction from the SAQ-UK, showed a trend towards improvement at month 1 for the Discussion arm (median 8 points) with a return to baseline levels by month 6, whilst the Pamphlet arm showed no change at either timepoint. For other secondary outcomes, the intervention arms showed similar trends over time. Tables for secondary outcome data are available in Additional file 4.

### Health economic data

Completion rates for resource use questions and EQ5D are summarised in Table 6. Health economic data will be published separately (Moure Fernández et al., in preparation).

## Discussion

An aim of the pilot was to provide information regarding the feasibility of conducting a large-scale trial in this patient population. Prior to the pilot, during the development of the interventions, patients received information about the study by letter and were asked to telephone a YTU researcher if they were interested in taking part. The response rate for this strategy was very poor and so it was altered for the pilot RCT. Rather than having actively to telephone a researcher, patients were asked by clinic staff on arrival at RACPC if they wanted to take part. This was much more successful, with an uptake rate for patients attending RACPC to recruitment into the pilot trial at around 40%.

We considered that the discussion intervention could improve reassurance in all patients attending RACPC, so patients with both positive (CHD) and negative (NCCP) test results were included in this pilot. Consistent with other studies, the majority of the patients recruited in RACPC received an NCCP diagnosis. Only 14% of patients were diagnosed with CHD so our numbers were too small to investigate effectiveness in the CHD subgroup separately. For example, we wished to investigate how satisfied patients receiving a CHD diagnosis were with their proposed treatment, measured by the treatment satisfaction scale within SAQ-UK, however this was not possible due to low numbers. Future trials would have to be considerably larger to recruit a reasonable number of CHD patients from this setting.

Return rates for the reassurance questionnaire were reasonable (80%) but many participants required one or more reminders. Up to three reminders were sent: reminder letters after 3 and 4 weeks with a final telephone reminder after 4 to 5 weeks. This led to some questionnaires being completed much later than planned. An earlier telephone reminder to encourage the return of primary outcome data should be considered for future trials. However, we do not think this altered our main outcomes following a sensitivity analysis including all patients with a questionnaire returned within a two week window either side of month 1.

Completion rates for other patient-reported questionnaires were lower (Guys and St Thomas’, HADS, BIPQ, SAQ-UK, chest pain questions and diaries). This was partly because patients’ responses to the reassurance questionnaire were collected during the reminder telephone call, but not the other questionnaires. Also, it is possible that the other questionnaires seemed less relevant to patients at month 1 and month 6, particularly if they were no longer experiencing chest pain. For example the BIPQ refers to the patient’s “illness”, whilst SAQ-UK and Guys and St Thomas’ questionnaire refer to chest pain or tightness. For future studies in this area, only the most relevant questionnaires should be included, focussing on those with high compliance. Additional instructions within the questionnaire could be included to improve completion rates.

This pilot study included investigations into the effectiveness of the discussion intervention, in terms of providing reassurance to patients about their test results in RACPC. According to a recent review “there is no generally accepted instrument to measure the level of reassurance” [23]. We used the 5-item instrument used in the NZ study. The questionnaire had not previously been validated in CHD patients, but we found it to be reliable and valid in our study population. In order to investigate whether Petrie’s categorisation of reassured/not reassured seemed appropriate, we looked at how patients answered question 3 (How reassured were you by the test? 0 represents ‘not at all’ and 10 represents ‘extremely reassured’; Additional file 5). A number of patients classed as ‘not reassured’ had high scores on this question (34 patients with score ≥ 8). A few patients answered 0 or 1 but are categorised as ‘reassured’. If the reassurance questionnaire was to be used in a future study, further investigation into how to classify patients into ‘reassured’ and ‘not reassured’ would be recommended.

We found no evidence of a difference in reassurance (both proportion of patients classed as “reassured” or mean score) between the discussion and pamphlet-only arms at month 1 or 6. The NZ study reported a significantly higher proportion of reassured patients in the discussion group (69%) compared to both the pamphlet-only group (40%) and a usual care control (35%) at month 1. Their results using mean reassurance score, however, were more similar to our pilot study; at month 1 the mean score was higher in the discussion group (43.4; 95% CI: 41.0 – 45.8) compared to both the pamphlet-only (38.4; 95% CI: 35.4 – 41.4) and control group (34.4; 95% CI: 30.5 – 38.4), but only the difference between discussion and control groups reached statistical significance. The NZ study was just in patients who received a negative (NCCP) test result, whereas this UK pilot also included patients diagnosed with CHD. Our sensitivity analyses on the NCCP subgroup showed a possible trend for higher reassurance in the Discussion arm (Additional file 6) but these differences were smaller than seen in the NZ study and not sustained at 6 months, and not considered to be clinically relevant.

The clinic setting for the studies was different, and it was likely that the experience for the patients in clinic was different. The UK pilot was conducted in a tertiary cardiology centre, with patients receiving more than one test (compared to just an ETT in the NZ study) and interaction with different staff for the different tests. It is possible that the usual care advice/information and explanation of test results in the UK RACPC was of a standard such that no additional benefit could be gained from a pre-test discussion. The discussion intervention was delivered by a research nurse who was not trained in psychological techniques and this may have contributed to the ineffectiveness of the discussion intervention. The target is to see UK patients in RACPC within 2 weeks of their GP visit, although this was not measured in this study, whereas patients had to wait longer for their clinic appointment in the NZ study (median 6 weeks). The NZ patients had more time to build up negative beliefs, which can influence reassurance in people with NCCP [13].

A limitation to this pilot was the absence of a third “no pre-test information” control arm. This was because Greater Manchester, where the study was conducted, had recommended that a Petrie-style pre-test pamphlet should be usual care in RACPC. As a result, we have no information on whether a pre-test pamphlet alone is better than no pre-test information in a UK setting. Since RACPCs are likely to send an information pamphlet with the appointment letter anyway, for example explaining what clothes to wear and what tests the patients may have in RACPC, it may be that RACPCs in regions other than Greater Manchester should consider including information regarding possible test results and ensuing treatments.

## Conclusions

An additional face-to-face discussion with a nurse, which re-iterated information given in a pre-test pamphlet, did not significantly improve reassurance in patients. Whilst there was a possible trend in the NCCP subgroup for improved reassurance with a discussion, the difference was not considered to be clinically important and does not warrant a larger trial of the discussion intervention in UK RACPCs.

## Electronic supplementary material

Additional file 1: **Pretest pamphlet.** All patients in the study received this pre-test pamphlet (A5, 4 pages) at the start of their RACPC appointment. (PDF 469 KB)

Additional file 2: **Responses to 5 reassurance questions.doc.** Plots of patients’ responses to the five questions (Questions 1 to 5) in the reassurance questionnaire at baseline (Questions 1 and 2 only), post-clinic, month 1 and month 6. Answers on a 0–10 scale. (DOCX 65 KB)

Additional file 3: **Chest pain diary data.** Table presenting patient-reported chest pain in a 7-day period at month 1 and month 6, collected from chest pain diaries. (DOCX 15 KB)

Additional file 4: **Secondary outcome data_HADS_BIPQ_SAQ-UK_Guys and St Thomas.** Three tables: (i) Change from baseline, at month 1 and month 6, for HADS, BIPQ and SAQ-UK; (ii) Individual item scores from the Brief Illness Perception Questionnaire at baseline, month 1 and month 6 and (iii) Guys and St Thomas’ chest pain score at baseline, month 1 and month 6. (DOCX 27 KB)

Additional file 5: **Reassurance Question 3.** Patients’ response to Question 3 of the Reassurance Questionnaire (How reassured were you by the test?) for those categorised as “reassured” and “not reassured” according to the method of Petrie et al., 2007. (DOCX 15 KB)

Additional file 6: **NCCP subgroup results.** Proportion of patients reassured and reassurance score at month 1 and month 6 for NCCP patients only. (DOCX 13 KB)

## Abbreviations

RACPC: Rapid access chest pain clinic
GP: General practitioner
CHD: Coronary heart disease
NCCP: Non-cardiac chest pain
RCT: Randomised controlled trial
NZ: New Zealand
NICE: National Institute for Health and Care Effectiveness
UHSM: University hospital of South Manchester
YTU: York trials unit
ETT: Exercise tolerance test
DSE: Dobutamine stress echocardiogram
HADS: Hospital anxiety and depression scale
BIPQ: Brief illness perception questionnaire
SAQ-UK: Seattle angina questionnaire - UK version.
