# Supplementary material for: An intervention to reassure patients about test results in rapid access chest pain clinic: a pilot randomised controlled trial
Source: BMC Cardiovasc Disord. 2014 Oct 4;14:138. doi: 10.1186/1471-2261-14-138 (PMC4197216; doi:10.1186/1471-2261-14-138)
Supplement: Supplementary file 1 — Additional file 1: Pretest pamphlet. All patients in the study received this pre-test pamphlet (A5, 4 pages) at the start of their RACPC appointment. (PDF 469 KB) [file 12872_2014_786_MOESM1_ESM.pdf]

Your chest pain results – what are the options?

At clinic there are three possible results:

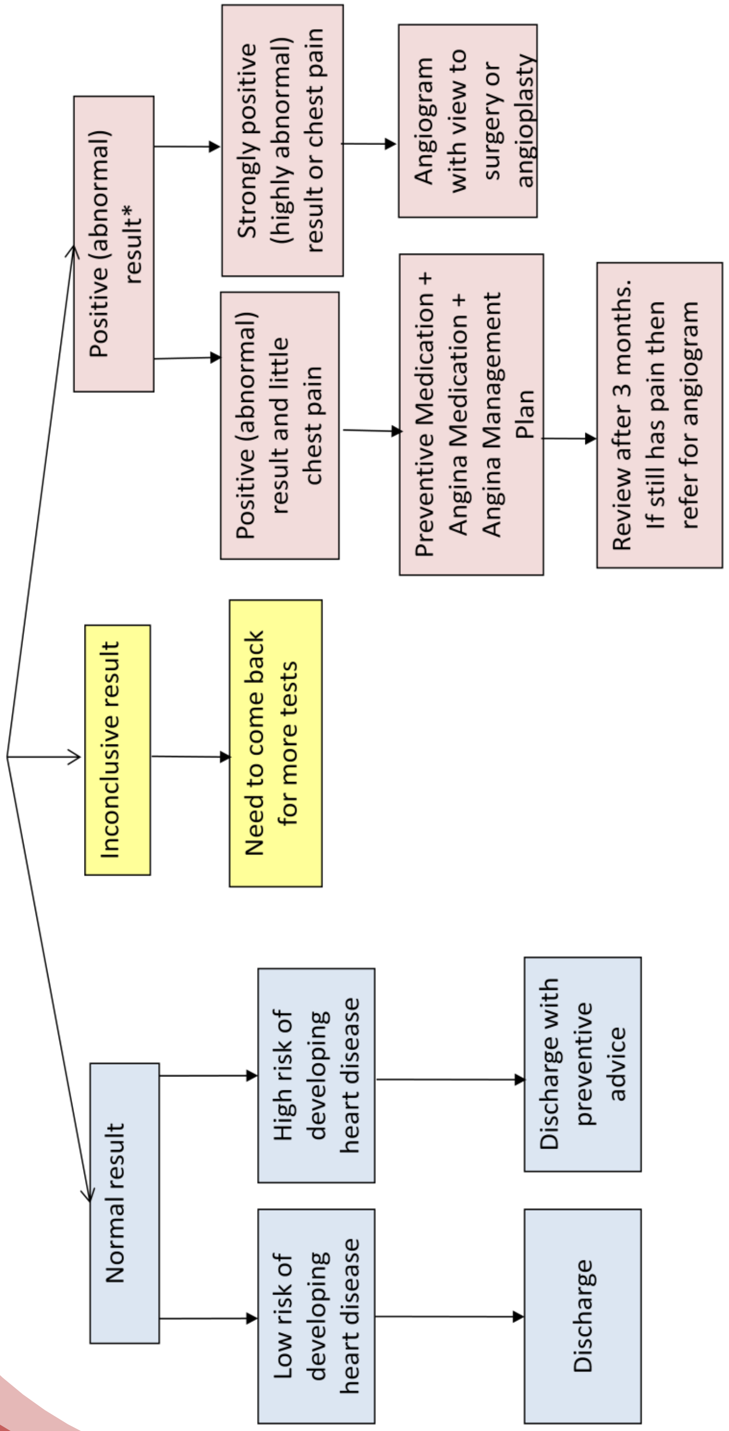

*A positive (abnormal) result means that you are likely to have heart disease

The Chest Pain Clinic

What do the results mean?

UHSM Your Hospital Working with Coventry University

The University of York The Department of Health Sciences

What could the test results mean?

There are three possible results.

- Normal – **half of people attending chest pain clinic will have a normal result,** some may need more preventive treatment.
- Inconclusive – more tests are needed that we cannot complete on the day of the appointment.
- Positive (abnormal) – If you are told that the result is positive or abnormal, it means that heart disease is likely. You may need further tests.

What happens if the result is positive (abnormal)?

There are two options:

1. **Strongly positive (highly abnormal) result OR chest discomfort on little activity**
   ⇒ You will be referred for an angiogram to decide on the most appropriate treatment.
2. **Positive (abnormal) result BUT little chest discomfort**
   ⇒ You will be prescribed medical treatment (medications to control chest pain + medications to reduce risk + Angina Plan angina management programme for lifestyle change).

Are medicines as good as surgery?

For people with a strongly positive or highly abnormal result or chest discomfort on little activity, angioplasty or surgery may be necessary to control the pain.

For people with a positive (abnormal) result and not much discomfort, then major trials have shown that medicines are as good as surgery or angioplasty in preventing heart attack or death.

What if they put me on medicines and I still get a lot of pain?

The doctors will try different combinations of medicines. If medication doesn’t control your chest discomfort in three months then you may be referred for an angiogram to see how best to treat you.

What if the results are normal?

When deciding how to treat you, it is not just the test results that matter. We also think about your risk factors for developing heart disease.

With a normal result there are two possibilities:

1. **Normal result + low risk of developing heart disease**
   ⇒ You would be referred back to your GP (your risk of developing heart disease is as low as the general public).
2. **Normal result + high risk of developing heart disease**
   ⇒ You would be referred back to your GP and given advice on lifestyle change to reduce your risk of having a heart attack and possible preventive treatment.

If the test is normal, what is causing my chest discomfort?

There are many causes of chest discomfort or chest pain – most of them have nothing to do with the heart, such as:

- Pain in the muscles between the ribs caused by tension in those muscles. Or pain in the chest wall caused by strains or tears of the muscles or ligaments.
- Spasm, tightening or mild inflammation of the oesophagus (the food pipe which runs through your chest between your throat and stomach). This pain is often caused by acid reflux from the stomach.

What if my chest discomfort continues but my result is normal?

- It is natural to be concerned or worried about what may be causing the discomfort. Concern or worry will often have other effects which contribute to the chest discomfort. These include muscle tension and breathing incorrectly.
- If your result is normal but you have ongoing chest discomfort that still worries you, a good person to talk to is your GP.

Overleaf is a diagram of the different treatment options. The doctor or nurse will discuss the best options for you after you have received your results.
