# Supplementary material for: An intervention to reassure patients about test results in rapid access chest pain clinic: a pilot randomised controlled trial
Source: BMC Cardiovasc Disord. 2014 Oct 4;14:138. doi: 10.1186/1471-2261-14-138 (PMC4197216; doi:10.1186/1471-2261-14-138)
Supplement: Supplementary file 3 — Additional file 3: Chest pain diary data. Table presenting patient-reported chest pain in a 7-day period at month 1 and month 6, collected from chest pain diaries. (DOCX 15 KB) [file 12872_2014_786_MOESM3_ESM.docx]

**Additional File 3: Data from a 7-day chest pain diary completed at month 1 and month 6**

From chest pain diaries, the average number of days with chest pain in a 7 day period was very similar at month 1 in the Discussion and Pamphlet arms (1.9 days vs 2 days) but at month 6 the Discussion arm had a higher average (1.2 days vs 0.6 days in the Pamphlet arm; see Table A below). A higher proportion of the Discussion arm, 20/42 (48%), reported zero days with chest pain at month 1 compared with 18/46 (39%) of the Pamphlet arm. At 6 months these proportions were 62% vs 73% respectively. Average episodes per day were similar between the arms at both timepoints.

## Table A – Patient-reported chest pain in a 7-day period at month 1 and month 6

|  | **Month 1** | | **Month 6** | |
| --- | --- | --- | --- | --- |
|  | **Discussion (n=42)** | **Pamphlet (n=46)** | **Discussion (n=37)** | **Pamphlet**  **(n=37)** |
| **Number of days with chest pain (Mean (SD))** | 1.9 (2.3) | 2.0 (2.1) | 1.2 (2.1) | 0.6 (1.2) |
| **Patients with no chest pain (N (%))** | 20 (47.6%) | 18 (39.1%) | 23 (62.2%) | 27 (73.0%) |

*SD* standard deviation
