# Supplementary material for: An intervention to reassure patients about test results in rapid access chest pain clinic: a pilot randomised controlled trial
Source: BMC Cardiovasc Disord. 2014 Oct 4;14:138. doi: 10.1186/1471-2261-14-138 (PMC4197216; doi:10.1186/1471-2261-14-138)
Supplement: Supplementary file 4 — Additional file 4: Secondary outcome data_HADS_BIPQ_SAQ-UK_Guys and St Thomas. Three tables: (i) Change from baseline, at month 1 and month 6, for HADS, BIPQ and SAQ-UK; (ii) Individual item scores from the Brief Illness Perception Questionnaire at baseline, month 1 and month 6 and (iii) Guys and St Thomas’ chest pain score at baseline, month 1 and month 6. (DOCX 27 KB) [file 12872_2014_786_MOESM4_ESM.docx]

**Additional File 4: Secondary outcome data - HADS, BIPQ, SAQ-UK and Guys and St Thomas**

The HADS anxiety and depression scores showed similar changes over time between the two intervention arms, with a reduction of less than 1 point in depression and 1.5 to 2 points reduction in anxiety score at month 6 (see Table B).

The individual BIPQ item scores (consequences, timeline, personal control, treatment control, identity, coherence, emotional and concern) were similar across the intervention arms (Table C). Cronbach’s alphas for an overall score were borderline (0.69 and 0.73 at months 1 and 6 respectively). The overall BIPQ score (Table B) reduced over time (indicating a less threatening view of their “illness”) in both arms by a median of 5 points at month 1 and 9 points at month 6. There were no differences between the intervention arms, however, change from baseline data were only available from approximately 50% of patients in the study.

Change from baseline in treatment satisfaction, from the SAQ-UK (Table B), showed improvement at month 1 for the Discussion arm (median 8 points) but a return to baseline levels by month 6. The Pamphlet arm showed no change at either timepoint (median 0 points change). Improvements in chest pain frequency and perception (SAQ-UK) were reported over time in both treatment arms, with greater improvement in the Pamphlet arm (median 27 and 33 points change in Discussion and Pamphlet arms, respectively, by month 6). Improvements in physical limitation score were seen in both groups across time.

The Guys and St Thomas’ questionnaire gives a score between 0 and 3 which has been shown to be predictive of coronary artery disease (0 is considered unequivocally ‘atypical’, 3 unequivocally ‘typical’ and 1 to 2 intermediate [Wu et. al. 2005]). Only two patients scored unequivocally typical at baseline (Table D). Whether score is predictive of a CHD diagnosis was not explored in this pilot.

## Table B: HADS, BIPQ and SAQ-UK: change from baseline at month 1 and month 6

| **Mean change from baseline (SD)** | **Month 1** | | **Month 6** | |
| --- | --- | --- | --- | --- |
|  | **Discussion** | **Pamphlet** | **Discussion** | **Pamphlet** |
| **HADS depression score** | -0.04 (2.8)  N=45 | -0.6 (2.2)  N=47 | -0.9 (2.6)  N=40 | -0.8 (3.0)  N=43 |
| **HADS anxiety score** | -1.0 (3.2)  N=45 | -1.0 (3.2)  N=47 | -1.8 (3.4)  N=40 | -1.5 (4.1)  N=43 |
| **Overall BIPQ score** | -6.6 (12.1)  N=30 | -7.1 (9.6)  N=33 | -12.2 (13.9)  N=25 | -11.3 (15.7)  N=30 |
| **SAQ-UK chest pain frequency and perception** | 19.4 (24.7)  N=44 | 21.6 (18.9)  N=47 | 24.5 (28.1)  N=38 | 31.5 (23.9)  N=41 |
| **SAQ-UK physical limitations** | 3.9 (13.4)  N=41 | 3.4 (11.8)  N=44 | 9.3 (26.5)  N=38 | 7.2 (16.9)  N=39 |
| **SAQ-UK treatment satisfaction** | 7.0 (33.5)  N=42 | 1.6 (24.8)  N=45 | 3.1 (37.0)  N=34 | 8.3 (23.9)  N=39 |

*SD* standard deviation

**Table C: BIPQ item scores at baseline, month 1 and month 6.** Higher scores represent a worse perception of the illness for consequences, timeline, identity, concern and emotional. Higher scores represent a better perception of the illness for personal control, treatment control and coherence.

|  | | **Baseline** | | **Month 1** | | **Month 6** | |
| --- | --- | --- | --- | --- | --- | --- | --- |
|  |  | **Discussion** | **Pamphlet** | **Discussion** | **Pamphlet** | **Discussion** | **Pamphlet** |
| **Consequences** | **Mean** | 4.43 | 3.65 | 3.36 | 2.87 | 2.45 | 1.64 |
|  | **Median** | 4.00 | 3.00 | 3.00 | 2.00 | 2.00 | 1.00 |
|  | **Std** | 2.58 | 2.70 | 2.62 | 2.62 | 2.33 | 2.11 |
|  | **StdErr** | 0.34 | 0.35 | 0.39 | 0.38 | 0.38 | 0.32 |
|  | **Max** | 10.00 | 10.00 | 10.00 | 10.00 | 8.00 | 7.00 |
|  | **Min** | 0.00 | 0.00 | 0.00 | 0.00 | 0.00 | 0.00 |
|  | **N** | 58 | 60 | 44 | 47 | 38 | 44 |
|  | **NMiss** | 2 | 0 | 16 | 13 | 22 | 16 |
| **Timeline** | **Mean** | 5.09 | 4.44 | 4.29 | 3.93 | 3.86 | 3.10 |
|  | **Median** | 5.00 | 5.00 | 4.50 | 3.00 | 3.00 | 2.00 |
|  | **Std** | 3.04 | 2.68 | 3.43 | 3.24 | 3.79 | 3.43 |
|  | **StdErr** | 0.41 | 0.37 | 0.53 | 0.48 | 0.63 | 0.55 |
|  | **Max** | 10.00 | 10.00 | 10.00 | 10.00 | 10.00 | 10.00 |
|  | **Min** | 0.00 | 0.00 | 0.00 | 0.00 | 0.00 | 0.00 |
|  | **N** | 54 | 54 | 42 | 45 | 36 | 39 |
|  | **NMiss** | 6 | 6 | 18 | 15 | 24 | 21 |
| **Personal control** | **Mean** | 3.90 | 3.58 | 3.98 | 5.00 | 5.78 | 5.73 |
|  | **Median** | 3.00 | 3.00 | 4.00 | 5.00 | 5.00 | 7.00 |
|  | **Std** | 2.70 | 3.11 | 3.14 | 3.41 | 3.31 | 3.63 |
|  | **StdErr** | 0.35 | 0.40 | 0.48 | 0.51 | 0.54 | 0.57 |
|  | **Max** | 10.00 | 10.00 | 10.00 | 10.00 | 10.00 | 10.00 |
|  | **Min** | 0.00 | 0.00 | 0.00 | 0.00 | 0.00 | 0.00 |
|  | **N** | 59 | 60 | 43 | 45 | 37 | 41 |
|  | **NMiss** | 1 | 0 | 17 | 15 | 23 | 19 |
| **Treatment control** | **Mean** | 7.29 | 6.73 | 4.87 | 5.58 | 4.24 | 5.24 |
|  | **Median** | 7.00 | 7.00 | 5.00 | 5.00 | 4.00 | 5.50 |
|  | **Std** | 2.12 | 2.81 | 3.53 | 3.41 | 3.50 | 3.90 |
|  | **StdErr** | 0.29 | 0.38 | 0.57 | 0.57 | 0.61 | 0.67 |
|  | **Max** | 10.00 | 10.00 | 10.00 | 10.00 | 10.00 | 10.00 |
|  | **Min** | 1.00 | 0.00 | 0.00 | 0.00 | 0.00 | 0.00 |
|  | **N** | 55 | 55 | 39 | 36 | 33 | 34 |
|  | **NMiss** | 5 | 5 | 21 | 24 | 27 | 26 |
| **Identity** | **Mean** | 5.20 | 4.83 | 3.24 | 3.14 | 2.27 | 1.95 |
|  | **Median** | 5.00 | 5.00 | 2.50 | 3.00 | 1.00 | 1.00 |
|  | **Std** | 2.49 | 2.63 | 2.55 | 2.61 | 2.29 | 2.11 |
|  | **StdErr** | 0.34 | 0.34 | 0.39 | 0.39 | 0.38 | 0.34 |
|  | **Max** | 10.00 | 10.00 | 9.00 | 10.00 | 7.00 | 7.00 |
|  | **Min** | 0.00 | 0.00 | 0.00 | 0.00 | 0.00 | 0.00 |
|  | **N** | 55 | 59 | 42 | 44 | 37 | 39 |
|  | **NMiss** | 5 | 1 | 18 | 16 | 23 | 21 |
| **Coherence** | **Mean** | 3.86 | 3.56 | 5.17 | 5.16 | 5.58 | 6.38 |
|  | **Median** | 4.00 | 3.00 | 5.00 | 5.00 | 5.50 | 8.00 |
|  | **Std** | 3.16 | 2.82 | 3.81 | 3.58 | 3.79 | 3.70 |
|  | **StdErr** | 0.41 | 0.37 | 0.59 | 0.54 | 0.63 | 0.59 |
|  | **Max** | 10.00 | 10.00 | 10.00 | 10.00 | 10.00 | 10.00 |
|  | **Min** | 0.00 | 0.00 | 0.00 | 0.00 | 0.00 | 0.00 |
|  | **N** | 59 | 57 | 42 | 44 | 36 | 39 |
|  | **NMiss** | 1 | 3 | 18 | 16 | 24 | 21 |
| **Emotional** | **Mean** | 5.66 | 4.60 | 4.34 | 3.31 | 3.74 | 2.43 |
|  | **Median** | 6.00 | 4.50 | 5.00 | 2.00 | 3.00 | 1.00 |
|  | **Std** | 2.84 | 3.34 | 2.99 | 3.21 | 3.14 | 3.02 |
|  | **StdErr** | 0.37 | 0.43 | 0.45 | 0.48 | 0.51 | 0.48 |
|  | **Max** | 10.00 | 10.00 | 10.00 | 10.00 | 9.00 | 10.00 |
|  | **Min** | 0.00 | 0.00 | 0.00 | 0.00 | 0.00 | 0.00 |
|  | **N** | 59 | 60 | 44 | 45 | 38 | 40 |
|  | **NMiss** | 1 | 0 | 16 | 15 | 22 | 20 |
| **Concern** | **Mean** | 6.28 | 5.77 | 4.52 | 4.39 | 3.03 | 3.20 |
|  | **Median** | 7.00 | 5.50 | 4.00 | 4.00 | 2.00 | 2.50 |
|  | **Std** | 2.77 | 2.68 | 3.12 | 3.07 | 3.02 | 3.07 |
|  | **StdErr** | 0.36 | 0.35 | 0.48 | 0.45 | 0.49 | 0.49 |
|  | **Max** | 10.00 | 10.00 | 10.00 | 10.00 | 10.00 | 10.00 |
|  | **Min** | 0.00 | 0.00 | 0.00 | 0.00 | 0.00 | 0.00 |
|  | **N** | 60 | 60 | 42 | 46 | 38 | 40 |
|  | **NMiss** | 0 | 0 | 18 | 14 | 22 | 20 |

**Table D: Guys and St Thomas’ score (0 to 3) by intervention group**

|  | **Discussion** | | | | | | **Pamphlet** | | | | | |
| --- | --- | --- | --- | --- | --- | --- | --- | --- | --- | --- | --- | --- |
|  | **Baseline** | | **Month 1** | | **Month 6** | | **Baseline** | | **Month 1** | | **Month 6** | |
|  | **N** | **%** | **N** | **%** | **N** | **%** | **N** | **%** | **N** | **%** | **N** | **%** |
|  | 14 | 23.33 | 22 | 46.81 | 32 | 68.09 | 15 | 25.00 | 19 | 38.00 | 33 | 64.71 |
| ***Missing data*** |  |  |  |  |  |  |  |  |  |  |  |  |
| **0** | 19 | 31.67 | 6 | 12.77 | 2 | 4.26 | 11 | 18.33 | 8 | 16.00 | 1 | 1.96 |
| **1** | 13 | 21.67 | 7 | 14.89 | 3 | 6.38 | 21 | 35.00 | 12 | 24.00 | 10 | 19.61 |
| **2** | 12 | 20.00 | 12 | 25.53 | 9 | 19.15 | 13 | 21.67 | 11 | 22.00 | 6 | 11.76 |
| **3** | 2 | 3.33 | . | . | 1 | 2.13 | . | . | . | . | 1 | 1.96 |
