# Supplementary material for: An intervention to reassure patients about test results in rapid access chest pain clinic: a pilot randomised controlled trial
Source: BMC Cardiovasc Disord. 2014 Oct 4;14:138. doi: 10.1186/1471-2261-14-138 (PMC4197216; doi:10.1186/1471-2261-14-138)
Supplement: Supplementary file 5 — Additional file 5: Reassurance Question 3. Patients’ response to Question 3 of the Reassurance Questionnaire (How reassured were you by the test?) for those categorised as “reassured” and “not reassured” according to the method of Petrie et al., 2007. (DOCX 15 KB) [file 12872_2014_786_MOESM5_ESM.docx]

Patients’ Responses to Reassurance Question 3

In order to investigate whether dividing patients using the median reassurance score into “reassured” or “not reassured” (as per Petrie et al., 2007) seemed an appropriate method of defining reassured patients the following table shows the number of patients answering question 3 (how reassured were you by the test) with each individual score from 0-10. 0 represents ‘Not at all’ and 10 represents ‘Extremely reassured’.

Data are shown for all patients at month 1 and month 6 for whom an overall reassurance score (and hence categorisation as reassured/not reassured) could be calculated (n=95 at month 1 and month 6).

It can be seen that there are a number of patients classed as ‘not reassured’ who have high scores on this specific question (34 patients with an 8 or above). There are also a few patients who answered 0 or 1 but are categorised as ‘reassured’.

|  | **Question 3. How reassured were you by the test?** | | | | | | | | | | |
| --- | --- | --- | --- | --- | --- | --- | --- | --- | --- | --- | --- |
|  | **0**  **Not at all** | **1** | **2** | **3** | **4** | **5** | **6** | **7** | **8** | **9** | **10**  **Extremely reassured** |
| **Reassured** | 1 | 2 | 0 | 0 | 0 | 1 | 2 | 2 | 12 | 22 | 57 |
| **Not reassured** | 5 | 2 | 7 | 6 | 4 | 12 | 7 | 14 | 19 | 7 | 8 |
