# Supplementary material for: An intervention to reassure patients about test results in rapid access chest pain clinic: a pilot randomised controlled trial
Source: BMC Cardiovasc Disord. 2014 Oct 4;14:138. doi: 10.1186/1471-2261-14-138 (PMC4197216; doi:10.1186/1471-2261-14-138)
Supplement: Supplementary file 6 — Additional file 6: NCCP subgroup results. Proportion of patients reassured and reassurance score at month 1 and month 6 for NCCP patients only. (DOCX 13 KB) [file 12872_2014_786_MOESM6_ESM.docx]

| **NCCP patient subgroup** | **Month 1** | | **Month 6** | |
| --- | --- | --- | --- | --- |
|  | **Discussion N=38** | **Pamphlet**  **N=38** | **Discussion N=36** | **Pamphlet**  **N=40** |
| % reassured (95% confidence interval) | 58% (42-74%) | 45% (29-61%) | 61% (45-77%) | 55% (40-70%) |
| Reassurance scores (least squares means accounting for baseline scores) | 35.8 (SE 0.81) | 34.2 (SE 0.80) | 37.7 (SE 0.83) | 35.1 (SE 0.79) |

*SE* Standard Error
