# Supplementary material for: An intervention to reassure patients about test results in rapid access chest pain clinic: a pilot randomised controlled trial
Source: BMC Cardiovasc Disord. 2014 Oct 4;14:138. doi: 10.1186/1471-2261-14-138 (PMC4197216; doi:10.1186/1471-2261-14-138)
Supplement: Supplementary file 6 [file 12872_2014_786_MOESM2_ESM.docx]

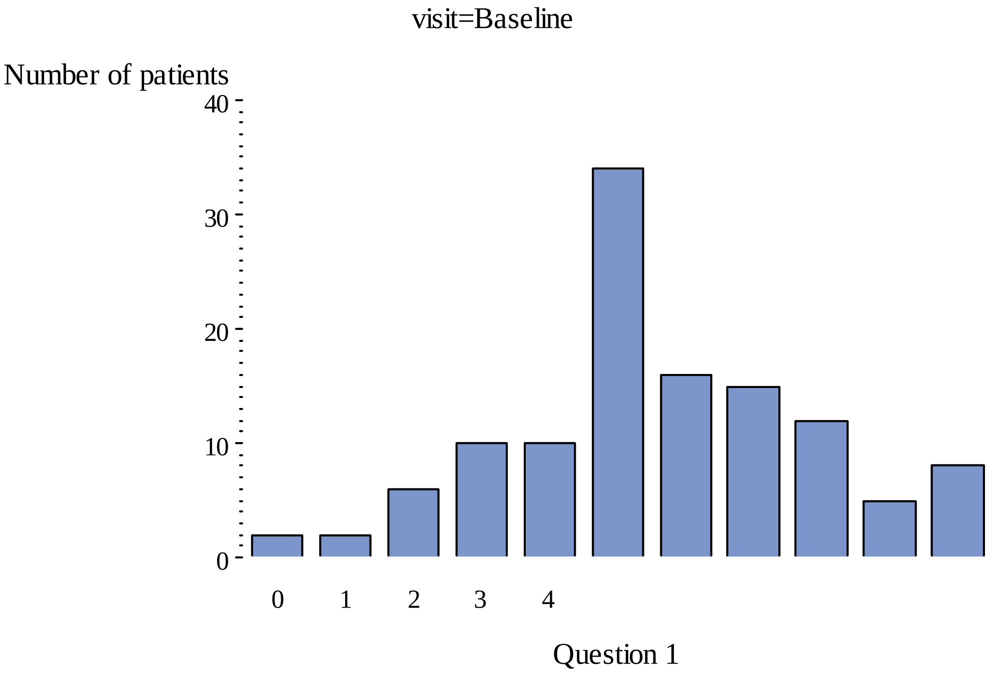
visit=Baseline
Number of patients
40
30
20
10
0
0
1
2
3
4
Question 1

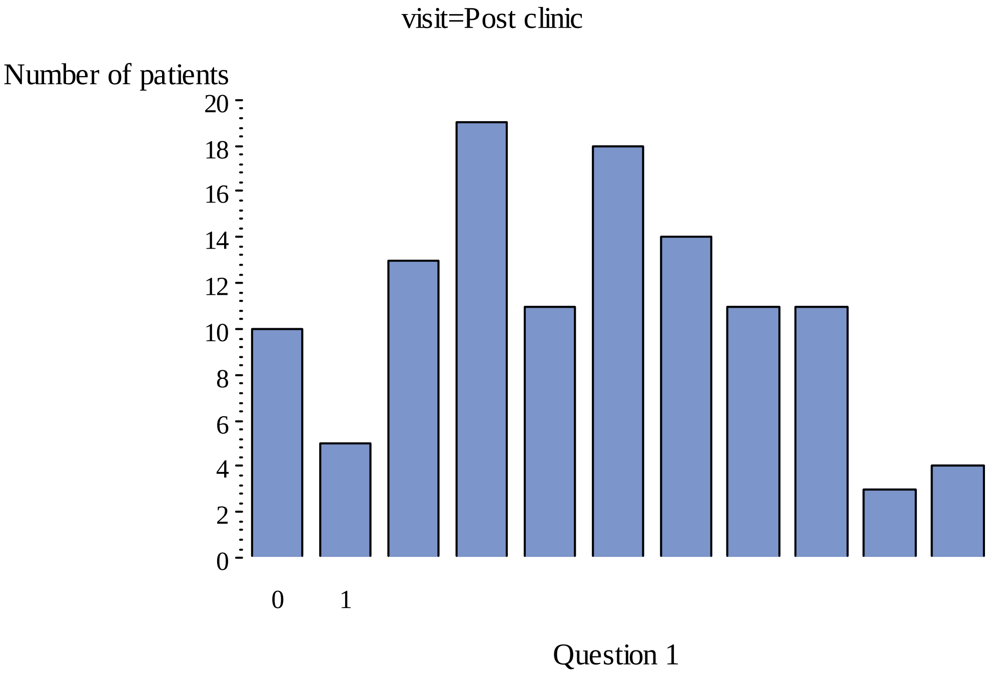
visit=Post clinic
Number of patients
20
18
16
14
12
10
8
6
4
2
0
0
1
Question 1

visit=Month 1

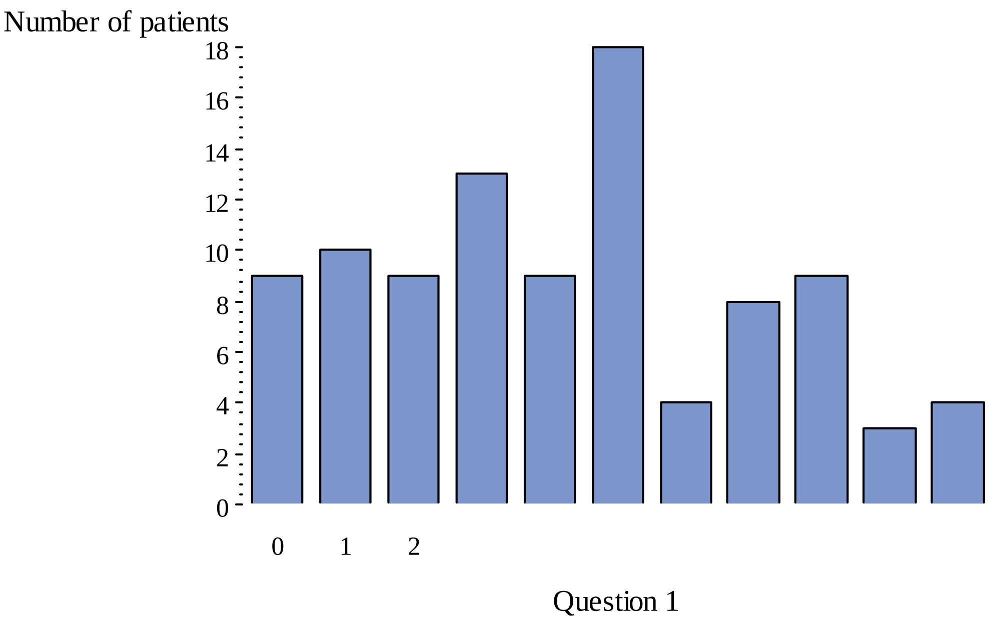

visit=Month 6

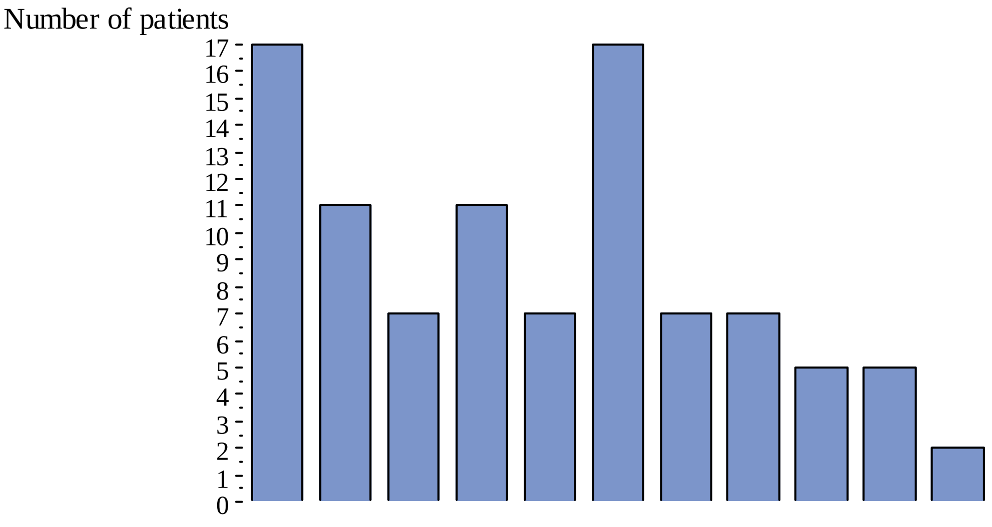

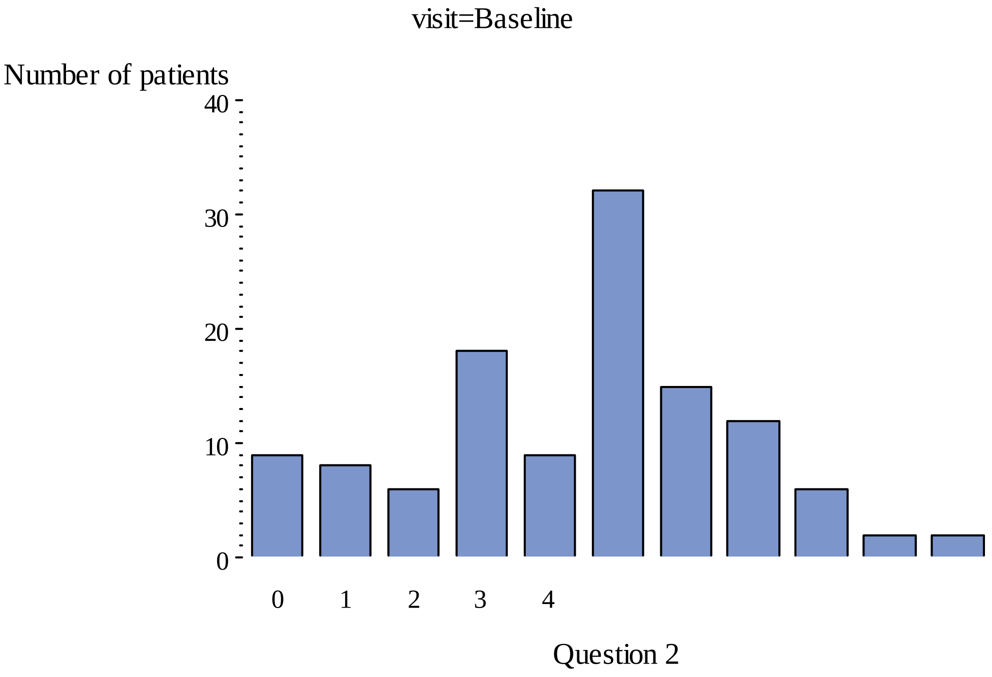
visit=Baseline
Number of patients
40
30
20
10
0
0
1
2
3
4
Question 2

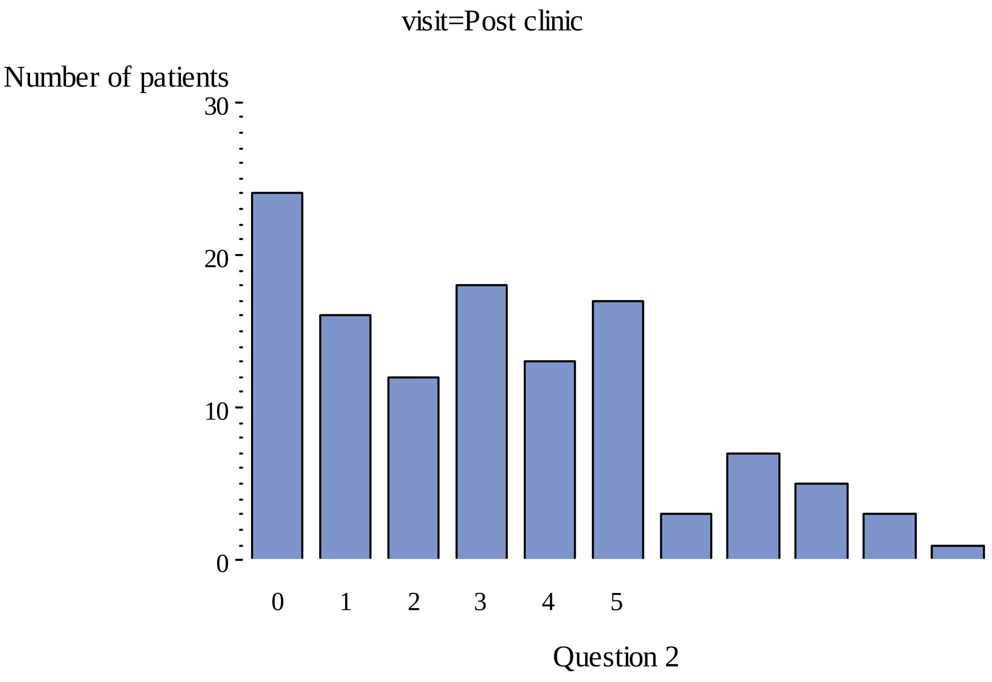
visit=Post clinic
Number of patients
30
20
10
0
0
1
2
3
4
5
Question 2

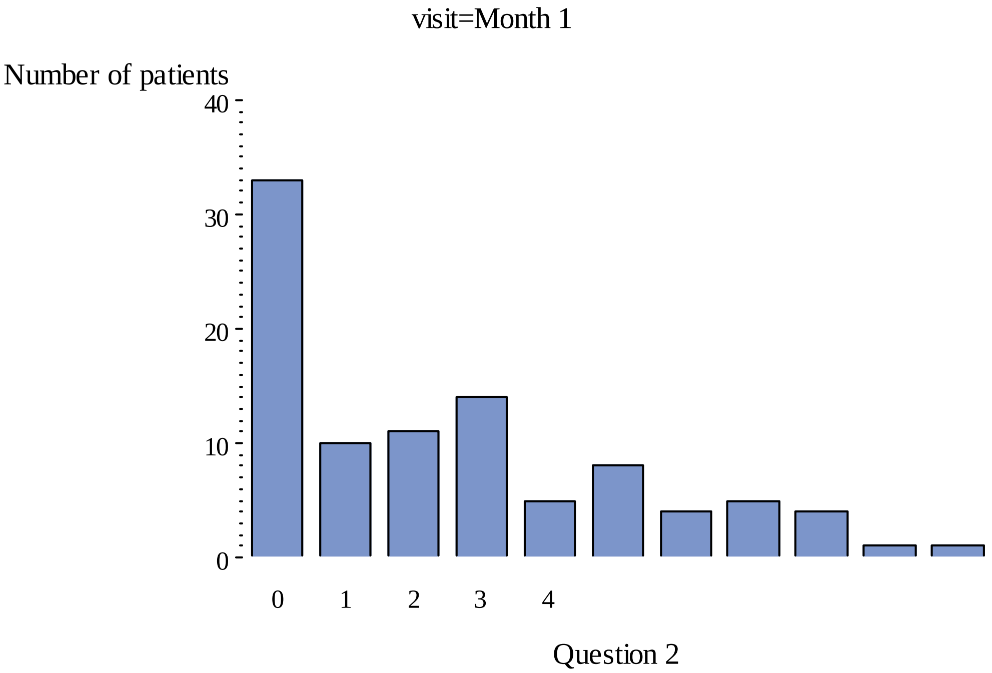
visit=Month 1
Number of patients
40
30
20
10
0
0
1
2
3
4
Question 2

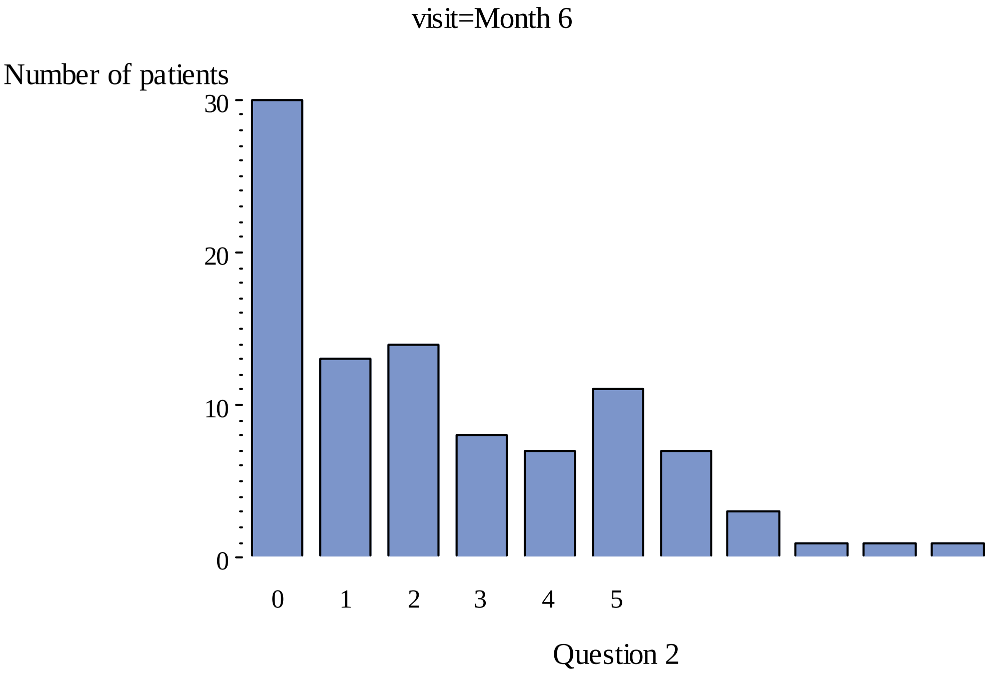
visit=Month 6
Number of patients
30
20
10
0
0
1
2
3
4
5
Question 2

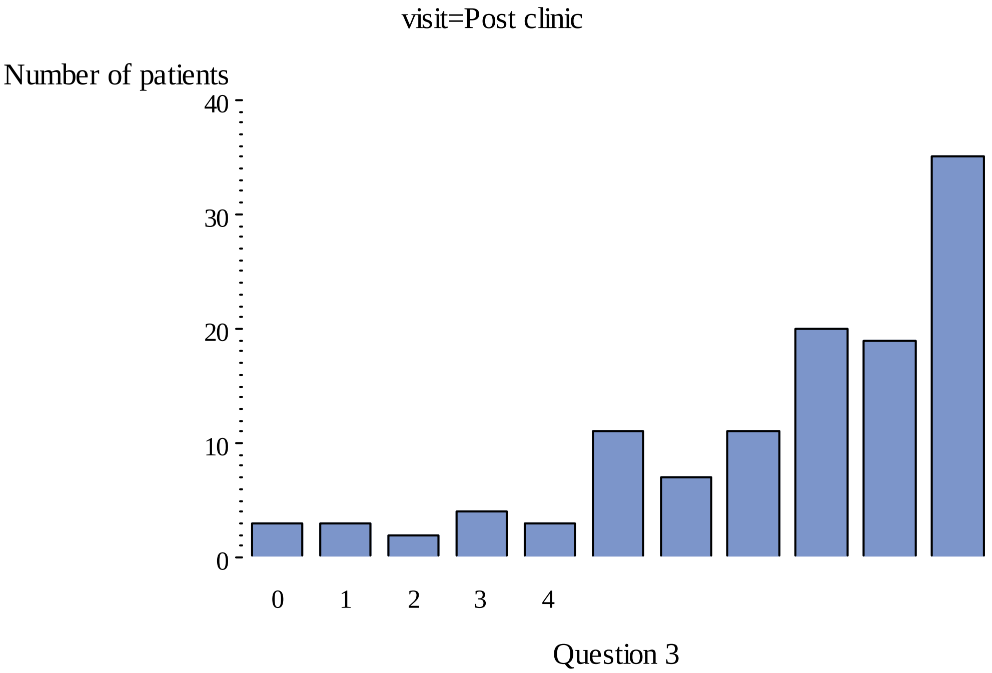
visit=Post clinic
Number of patients
40
30
20
10
0
0
1
2
3
4
Question 3

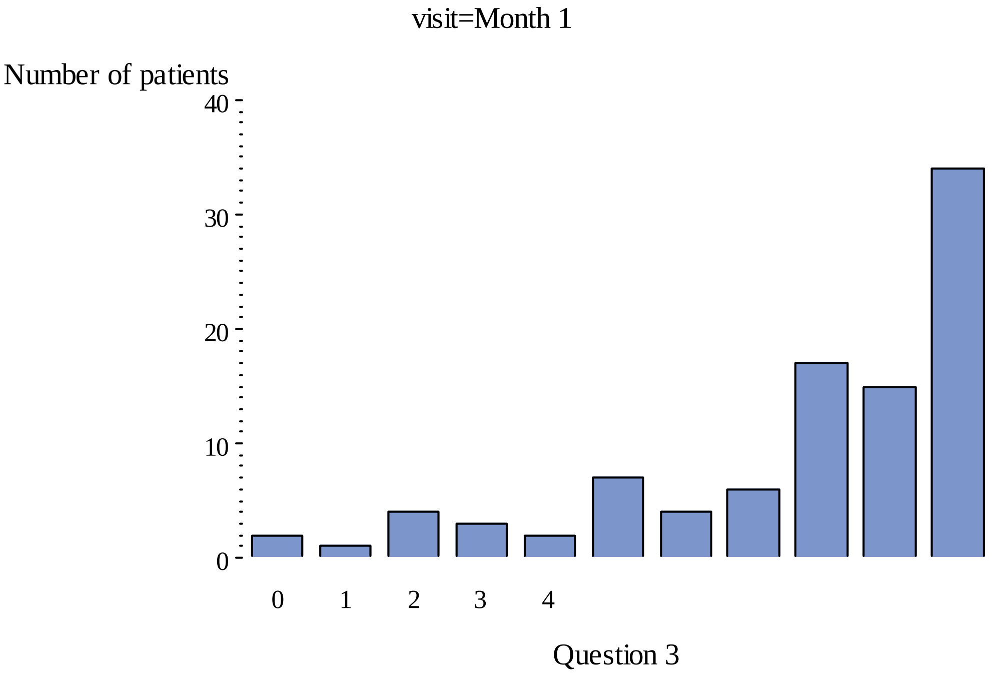
visit=Month 1
Number of patients
40
30
20
10
0
0
1
2
3
4
Question 3

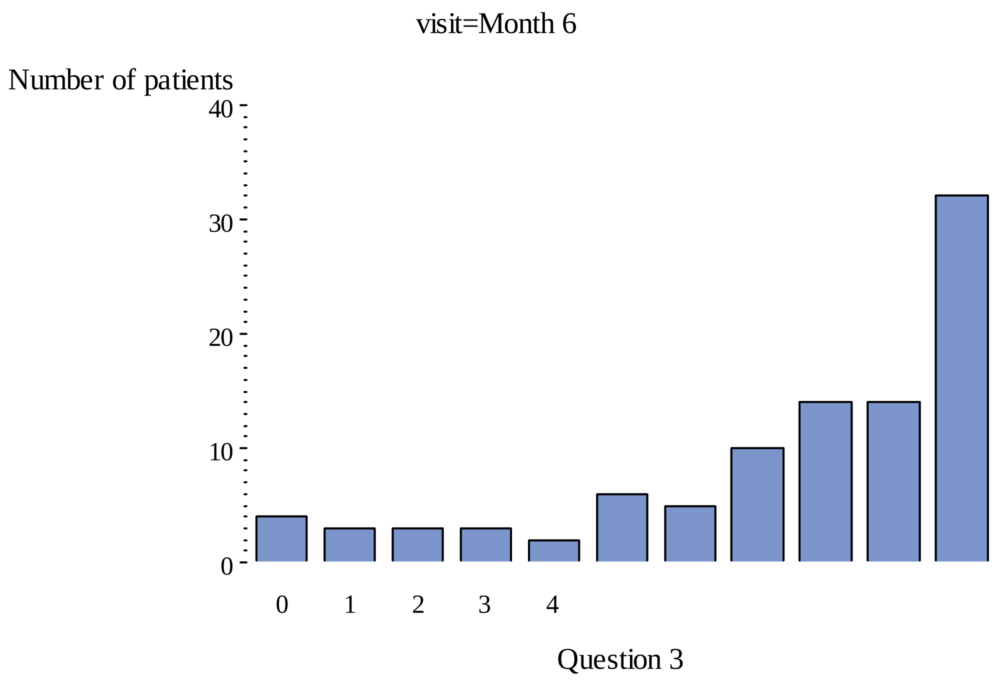
visit=Month 6
Number of patients
40
30
20
10
0
0
1
2
3
4
Question 3

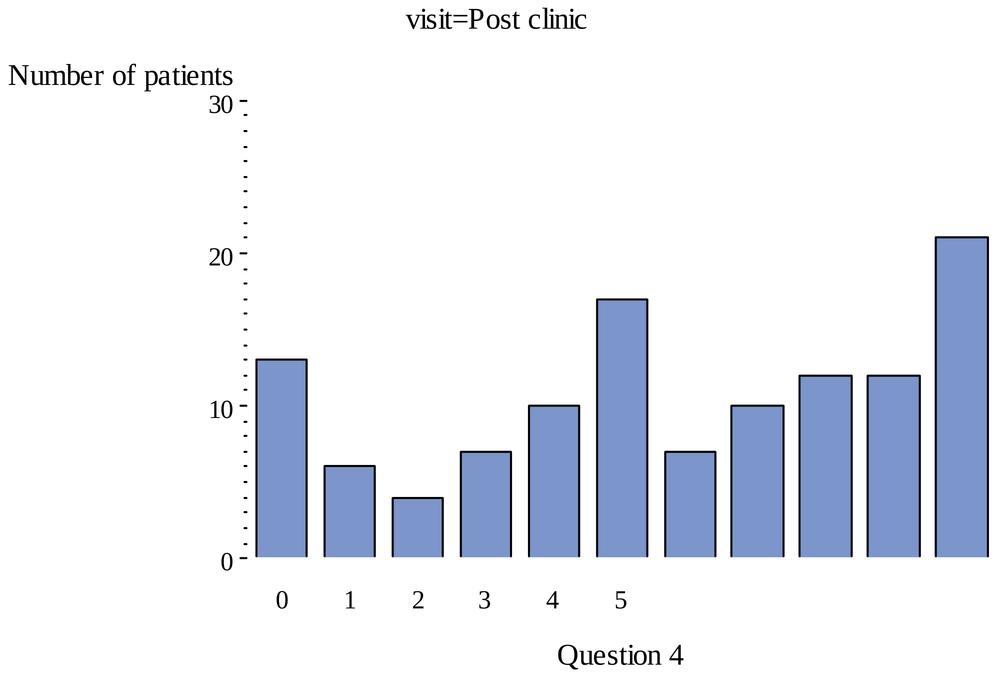
visit=Post clinic
Number of patients
30
20
10
0
0
1
2
3
4
5
Question 4

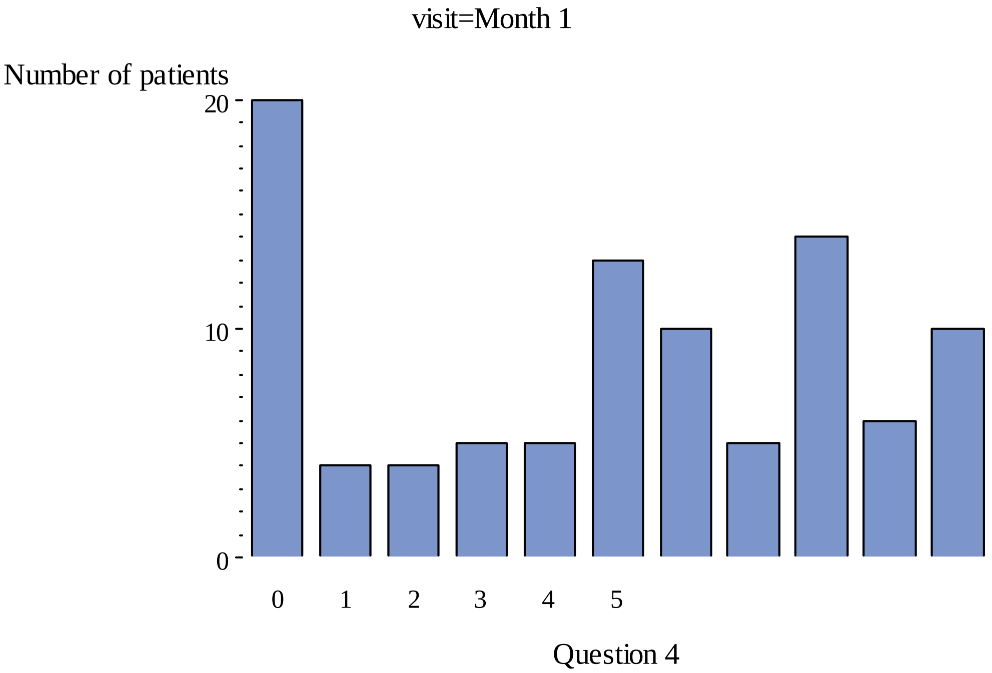
visit=Month 1
Number of patients
20
10
0
0
1
2
3
4
5
Question 4

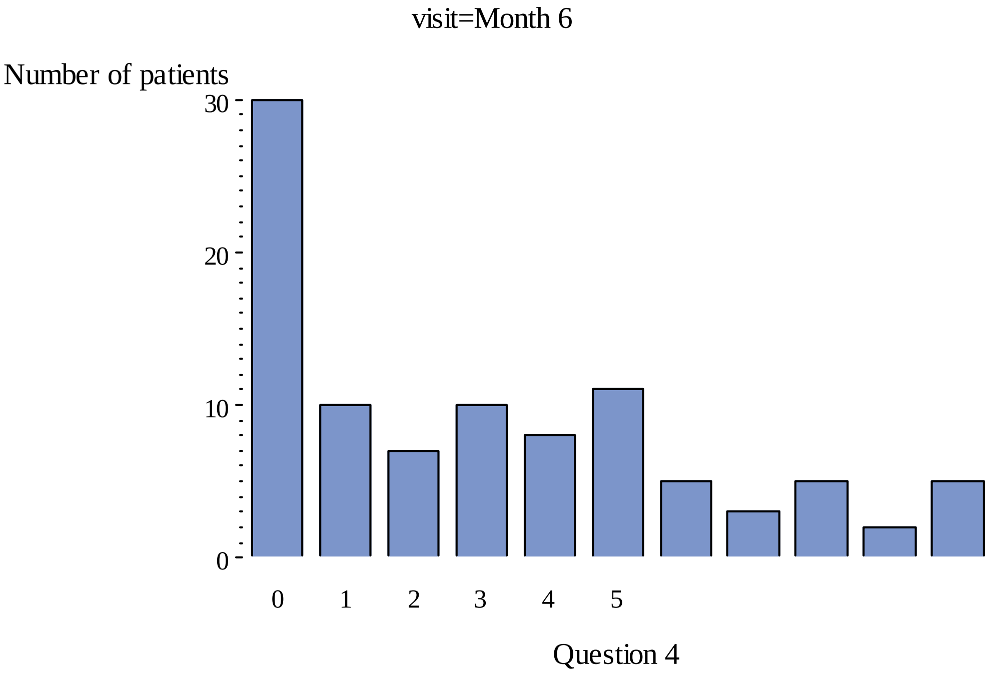
visit=Month 6
Number of patients
30
20
10
0
0
1
2
3
4
5
Question 4

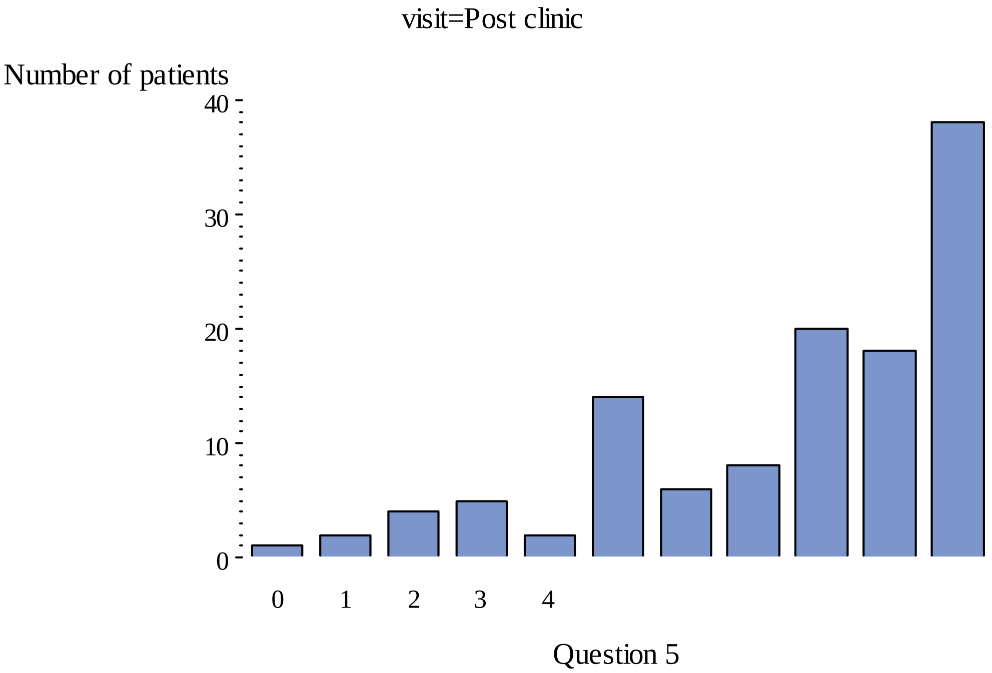
visit=Post clinic
Number of patients
40
30
20
10
0
0
1
2
3
4
Question 5

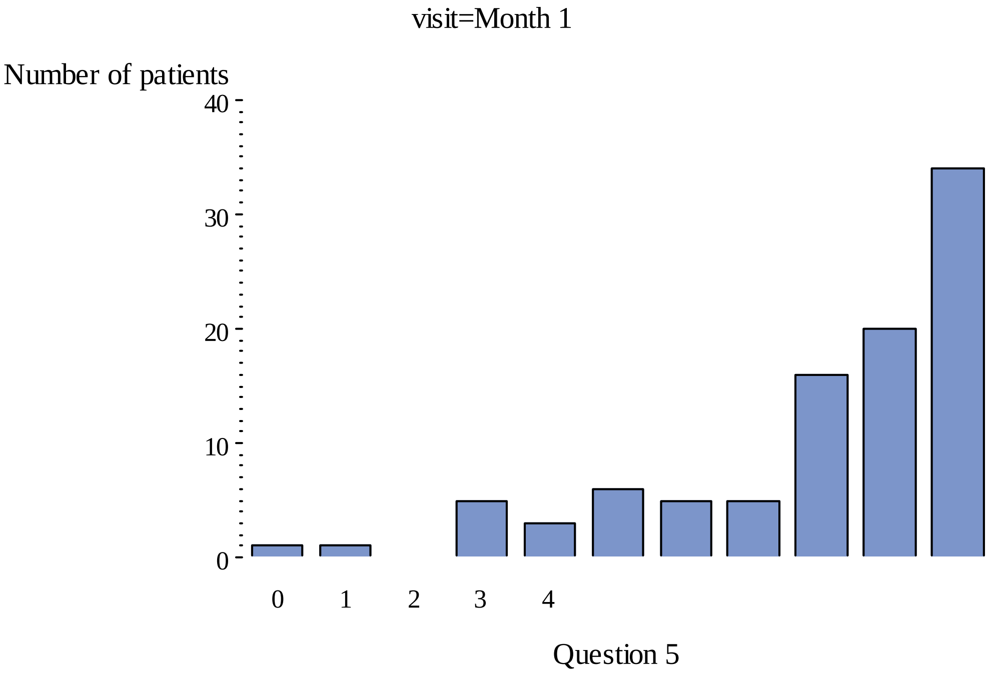
visit=Month 1
Number of patients
40
30
20
10
0
0
1
2
3
4
Question 5

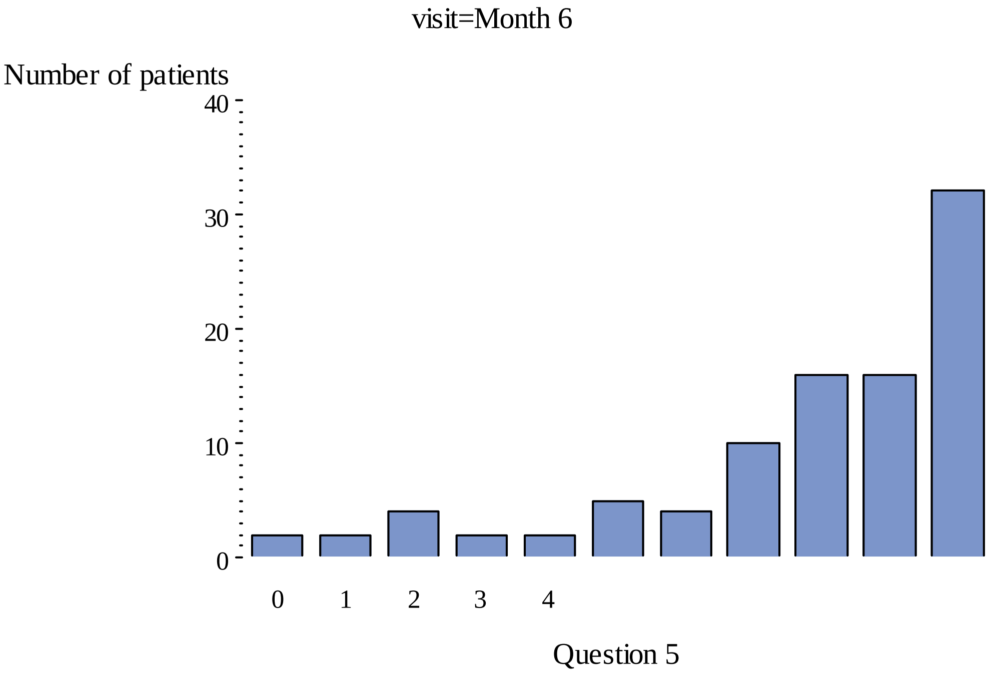
visit=Month 6
Number of patients
40
30
20
10
0
0
1
2
3
4
Question 5
